# Cytokine profile during occult hepatitis B virus infection in chronic hepatitis C patients

**DOI:** 10.1186/s12985-021-01487-2

**Published:** 2021-01-12

**Authors:** Camilla Rodrigues de Almeida Ribeiro, Nathalia Alves Araújo de Almeida, Katrini Guidolini Martinelli, Marcia Amendola Pires, Carlos Eduardo Brandao Mello, José J. Barros, Vanessa Salete de Paula

**Affiliations:** 1 Laboratory of Molecular Virology, Oswaldo Cruz Institute, Oswaldo Cruz Foundation, 4365, Brasil Av., Manguinhos, Rio de Janeiro, RJ 21040-360 Brazil; 2 Federal University of Espírito Santo, Espírito Santo, Brazil; 3 Gaffrée and Guinle Universitary Hospital, Ambulatory of Liver Disease, Rio de Janeiro State Federal University/UniRio, Rio de Janeiro, Brazil

**Keywords:** Occult hepatitis B infection, Cytokines, HBV/HCV co-infection

## Abstract

**Background:**

The hepatitis B virus (HBV) is one of the leading causes of acute, chronic and occult hepatitis (OBI) representing a serious public health threat. Cytokines are known to be important chemical mediators that regulate the differentiation, proliferation and function of immune cells. Accumulating evidence indicate that the inadequate immune responses are responsible for HBV persistency. The aim of this study were to investigate the cytokines IFN-γ, TNF-α, IL-2, IL-4, IL-6, IL-10 and IL-17A in patients with OBI and verify if there is an association between the levels of these cytokines with the determination of clinical courses during HBV occult infection.

**Methods:**

114 patients with chronic hepatitis C were investigated through serological and molecular tests, the OBI coinfected patients were subjected to the test for cytokines using the commercial human CBA kit. As controls, ten healthy donors with no history of liver disease and 10 chronic HBV monoinfected patients of similar age to OBI patients were selected.

**Results:**

Among 114 HCV patients investigated, 11 individuals had occult hepatitis B. The levels of cytokines were heterogeneous between the groups, most of the cytokines showed higher levels of production detection among OBI/HCV individuals when compared to control group and HBV monoinfected pacients. We found a high level of IL-17A in the HBV monoinfected group, high levels of TNF-α, IL-10, IL-6, IL-4 and IL-2 in OBI/HCV patients.

**Conclusion:**

These cytokines could be involved in the persistence of HBV DNA in hepatocytes triggers a constant immune response, inducing continuous liver inflammation, which can accelerate liver damage and favor the development of liver cirrhosis in other chronic liver diseases.

## Background

Hepatitis B virus (HBV) affects 2 billion people worldwide. Of these, approximately 350 million have a chronic infection with a risk of developing a serious condition, with cirrhosis and hepatocellular carcinoma (HCC), which cause 500 to 700 thousand deaths per year in the world [1, 2]. The disease caused by the HBV can result in asymptomatic infection, acute self-limiting hepatitis, chronic hepatitis, fulminant hepatitis, occult HBV infection (OBI) and in more severe cases requires liver transplantation [3].

The persistence of replication-competent HBV DNA (i.e. episomal HBV covalently closed circular DNA [cccDNA]) in the liver tissue and/or blood of patients with negative results for the hepatitis B surface antigen (HBsAg) antigen by currently available assays is called occult HBV infection [4–6]. It can occur after the resolution of a self-limited acute infection or after a long time of infection, if there is any clinical evidence or biochemical change in liver function, using these virus carriers, with a potential risk of transmitting the infection [5, 7, 8]. In this context, the antibody against the viral core (anti-HBc) should be considered to investigating patients for OBI [9, 10].

There are three types of OBI: seropositive, seronegative and a case called “false”. Seropositive OBI is characterized by the detection of anti-HBc antibody with or without anti-HBs. The OBI seronegative is characterized by undetectable antibodies both anti-HBc and anti-HBs. OBI seropositive is responsible for the vast majority of OBI cases, which can be attributed to the higher proportion of resolved HBV infections. However, more than 20% of individuals with OBI do not have serological markers, either due to the drop-in antibody titers over time that become undetectable, or because there has never been seroconversion, this latter case is known as OBI seronegative [11]. Thus, OBI can be found both in seropositive individuals (with the presence of anti-HBc accompanied or not by anti-HBs), and in seronegatives, making HBV-DNA the only marker of HBV infection, detectable at low levels (< 200 IU/mL) [5]. "False" OBI occurs due to the presence of mutations in the S gene (escape mutants) that produce modified HBsAg that are not recognized by commercially available detection assays [11–13].

Although the clinical implications are almost minimal in patients with OBI, the biggest concern is the fact that transmission to healthy people even with very low viral load values. There is also the possibility or ability to reactivate viral replication in the presence immunosuppression, which can lead to severe acute conditions and liver decompensation with high mortality [6, 14].

The mechanisms involved in the pathogenesis of OBI have been the subject of several studies, but they still need to be clarified. The pathogenesis of OBI can be multifactorial depending largely on the virus-host interaction, mediated by the immune response [15]. Several evidences demonstrate that the virus-host interactions are related to the induction and maintenance of the occult phase of infection by the hepatitis B virus. The host's immune response is linked to viral persistence and the immunopathogenesis of the infection [16]. Concomitant hepatitis C virus (HCV) infection and other risk factors, such as alcohol consumption, are also associated with occult HBV infection that can progress to chronic liver disease [17]. HBV reactivation has been reported in patients with chronic HCV infection under treatment with more recent direct-acting antivirals (DAA), resulting in fulminant hepatitis, liver failure and, in some cases, death [18].

The prevalence of OBI among patients with chronic hepatitis C (CHC) varies widely from 0 to 52% [19]. HBV can maintain its oncogenic potential in all clinical situations of the course of its infection, including OBI and it is estimated that the existence of other causes that lead to liver damage, such as HCV co-infection, accelerate this process [20]. Some studies indicate that OBI unfavorably affects the progression of liver fibrosis and the development of HCC in patients with CHC [21–23].

There are clear differences in adaptive immunity in patients with chronic or resolved HBV infection. The responses of HBV-specific CD4+ and CD8+ T cells with a Th1 cytokine production profile are detectable in high blood titers in individuals with a favorable outcome. These Th1 cell responses along with cytotoxic responses are quantitatively stronger than those found in patients with chronic infections, which are, on the contrary, characterized by weaker or undetectable T cell responses to the virus [24–27].

Differences between the host's immune response may be one of the reasons for the development and progression of hepatitis B [28, 29]. Cytokines represent a large family of molecules, including cytokines associated with type 1 (Th1) T cell responses (eg, interleukin (IL-2), interferon (IFN-γ)), which have a functional contribution to cell immune responses; type 2 (Th2) T cell response (eg, IL-4, IL-6, IL-10), which play a role in humoral immune responses; cytokines associated with regulatory T cells (Treg) with, for example, IL-10 which is associated with immunomodulation and immunosuppression; and T-cell response of type 17 (Th17) (for example, IL-17), which play critical roles in mediating inflammation [30].

Here we investigated the cytokines IFN-γ, TNF-α, IL-4, IL-6, IL-10 and IL-17A that have a great impact on the clinical outcomes associated with hepatitis B. However, this impact is still not well understood and some cytokines such as IFN-Y, IL-6, IL-2 and IL-4 have a controversial role in HBV infection, which may induce cirrhosis and HCC or eliminate HBV via inducing humoral and cellular immune response [28, 31–33]. Cytokines such TNF-α, IL-10 and IL-17A are well described in association with chronic hepatitis, cirrhosis and HCC in combination with other proinflammation cytokines [34–36].

The aim of this study was to investigate the presence of pro and anti-inflammatory cytokines IFN-γ, TNF-α, IL-2, IL-4, IL-6, IL-10 and IL-17A in patients with occult hepatitis B and to verify if there is an association between the levels of these cytokines with the determination of clinical courses during HBV occult infection.

## Materials and methods

### Population Studied

This is a retrospective cross-sectional study conducted in patients prior to commencement of DAA treatment who had attended the Outpatient Clinic of Liver Disease at the Gaffrée and Guinle University Hospital (Rio de Janeiro, Brazil) from January to December 2018. All patients enrolled into the study signed an informed consent form after being provided all necessary information to make an informed decision. Socio-epidemiological data, information about infection, HBV treatment and risk behaviors were obtained from each patient record or from the questionnaire.

Serum samples were collected from a cohort of 114 consecutive HCV patients. All serum samples were HCV RNA-positive and examined for total anti-HBc and HBsAg via immunoenzymatic assays (EIA). Samples positive for anti-HBc with no HBsAg indicating seropositive OBI were further examined using real-time PCR (qPCR) and nested PCR [37]. The HBV DNA was additionally sequenced for genotyping, HBV genotyping was performed by phylogenetic analyses of the pre-S/S gene with HBV sequences representing all genotypes available in GenBank. Phylogenetic analysis was performed using the maximum likelihood method with the online version of the PhyML program [38]. The reliability of the phylogenies was estimated with the approximate likelihood-ratio test [39] based on a Shimodaira-Hasegawa-like procedure (SH-aLRT).

Occult infections were considered as those with viral DNA in the serum tissue of HBsAg-negative individuals. Of the 114 HCV patients, 11 individuals had occult hepatitis B and were included in this study. As controls, ten healthy donors with no history of liver disease and 10 chronic HBV monoinfected patients of similar age to OBI/HCV patients were selected.

### Biochemical tests

Serum samples were subjected to biochemical doses of liver enzymes, such as aspartate aminotransferase (AST); alanine aminotransferase (ALT); alkaline phosphatase; total, direct and indirect bilirubin; and gamma-glutamyl transferase (GGT) through a system of quantitative determination by photometry in kinetic mode using a commercial kit (LabMax 560, LabTest, Minas Gerais, Brazil) according manufacture instructions.

### Cytokine quantification

Peripheral blood obtained was centrifuged on 800 g for 10 min at room temperature. Sera was collected and stored at − 70 °C until test performance. The serum levels of IL-2, IL-4, IL-6, IL-10, TNF-α, IFN-γ, and IL17A were determined by means of flow cytometry with a BD Cytometric Bead Array (CBA) Human Th1/Th2/Th17 Cytokine Kit (BD Biosciences San Jose, CA), following the manufacturer’s technical guidelines and protocols. A FACS Canto II flow cytometer (BD Biosciences, NJ, USA) was used for sample acquisition, and FCAP-Array software (ver. 3.0.1) was used to calculate cytokine levels and the mean fluorescence intensity of each cytokine.

### Data analysis

The SPSS 2.0 program was used to perform the statistical analyzes. Descriptive statistics of the qualitative variables was determined by frequency distribution and quantitative variables by median and P25-75. Afterwards, the normality of data distribution was assessed by the Kolmogorov–Smirnov test. The Mann–Whitney nonparametric test was used to compare cytokine levels between the groups. Correlation was estimated by Spearman test (the data did not present a normal distribution). Values of *p* ≤ 0.05 and 95% confidence intervals (CIs) were considered as significant for all statistical analysis. Graphs were built using Graphpad 5.0 (Graphpad software, San Diego, CA, USA).

### Ethical approval

The study protocol was approved by the Research Ethics Committee of the Institute Oswaldo Cruz (CAAE 34246914.4.1001.5248 number 2.927.747/18).

## Results

### Sociodemographic and bioclinical characteristics of the study population

Occult HBV infection was detected in eleven patients (9,64%). The majority of the OBI patients was woman (54.5%) with a mean age of 65.17 ± 9.57. The patients had received treatment with pegylated interferon (80 mcg) and ribavirin (1.0 g per day) for 24 weeks without HCV clearance. No patients developed ALT and AST flareups and all were anti-HB-positive, with viral loads ranging from 207.14 to 266,114.8 IU/mL (8.5 × 10^2^ to 1.49 × 10^8^ copies/mL). Patients with the highest viral loads were positive for S-region amplification via nested-polymerase chain reaction (nested-PCR) and HBV in these cases was classified as genotype A (A1 and A2 subtypes) (Additional file 1: Table 1).

**Table 1:** Demographic and clinical data in patients infected with HCV and occult hepatitis B. Rio de Janeiro, 2018

Categorical variables	N	%
Gender		
Female	06	54.5
Male	05	45.5
Fibrosis stage		
F2	02	18.2
F4	09	81.8
Genotype HCV		
1a	05	45.5
1b	04	36.4
3a	02	18.2
Genotype HBV		
A1	01	9.1
A2	02	18.2
ND	08	72.7

Continuos variables	Mean	SD
Age (years)	65.64	6.89
Viral Load (log)	3.36	1.01
ALT*	62.91	29.92
AST*	72.09	41.95
GGT*	76.64	64.02
BT*	0.67	0.27
Copies/mL—HBV	1.76E + 07	4.56E + 07
IU/mL**	32,851.90	80,988.23

Continuos variables	Median	P25–75
Age (years)	66	62–69
Viral Load (log)	3.26	2.61–3.71
ALT*	68	39–76
AST*	71	33–109
GGT*	65	38–94
BT*	0.80	0.40–0.90
Copies/mL—HBV	1.03E + 04	2.26E + 03–8.50E + 04
IU/ml**	1839.5	403–5178.1

*SD* standard deviation, *ND* not determined, *P25* 25th percentile, *P75* 75th percentile

*AST (alanine aminotransferase)—Reference values in chronic hepatitis: < 31 U/L (women) and 37 U/L (men); *ALT (alkaline phosphatase)—Reference values in chronic hepatitis: < 31 U/L (women) and < 41 U/L (men); *GGT (gamma-glutamyl transferase) – Reference values 8–61 U/L (men) and 5–36 U/L (women); *BT (total **1 copies/ml. corresponds to 5.26 IU/ml)

Nine patients had chronic active hepatitis C, grade 4, with cirrhosis (F4, 14.1–46.4 kPa; fibroscan), and two patients had chronic active hepatitis C, grade 2, with cirrhosis (F2, 6.8–6.9 kPa; fibroscan). After the study, all patients were re-treated with sofosbuvir (400 mg) + daclatasvir (60 mg) + ribavirin (1.0 g), following which HCV remained undetectable. Information on gender, age distribution, viral load and genotype distribution of the OBI patients are described in Table 1. No correlation was found between liver enzymes (ALT and AST), the patients age and viral load of HBV or HCV.

### Expression levels of cytokines

Although, most of the patients were female and belonged to HCV genotype 1a no correlation was found in the cytokine levels, gender, liver enzymes (ALT and AST), age and HBV or HCV viral load.

In the present study, a significant difference was found in the medians, we found higher medians of IL-17-A in the HBV monoinfected group; a significant difference was found for IL-17A (*p* = 0,0317) between the OBI/HCV and HBV monoinfected groups.

TNF-α (*p* = 0.020), IL-6 (*p* < 0.0001), IL-4 (*p* = 0.024) and IL-2 (*p* = 0.043) were higher in the OBI/HCV group and no significant difference was found for INF-Y (*p* = 0.512) and IL-10 (*p* = 0.114) compared to the controls. A significant difference was found for IL-6 (*p* = 0,0014) between the OBI/HCV and HBV monoinfected groups and no significant difference was found between HBV monoinfected and controls for all analyzed cytokines (Table 2).

**Table 2:** Median values (pg/mL) of cytokines in each group

Citokyne	Control		OBI/HCV			HBV	
	Median	P25–P75	Median	P25–P75	Median		P25–P75
IL-17A	10.36^a^	7.92–12.67	6.38^a,b^	1.34–8.26	10.91^b^		6.54–16.43
INF-Y	0.43	0.0–0.65	0.43	0.0–5.32	0.05		0.0–0.23
TNF-α	0.0^a^	0.0–0.99	2.77^a^	1.02–24.80	0.00		0.00–13.74
IL-10	0.25	0.12–0.56	2.38	0.0–2.74	0.58		0.01–3.40
IL-6	0.67^a^	0.43–1.76	9.76^a,b^	7.51–23.33	1.43^b^		0.76–4.18
IL-4	2.22^a^	1.54–2.97	6.14^a^	1.87–20.98	1.82		1.43–13.00
IL-2	0.0^a^	0.0–1.11	1.22^a^	0.0–8.64	0.06		0.0–8.23

25th percentile (P25) and 75th percentile (P75) according to different levels of cytokines. To know the difference between the groups, the Mann–Whitney nonparametric test was used. Equal letters show groups with statistically significant mean difference

The mean values of IL-6 (*p* = 0.025) and IL-4 (*p* = 0.045) were higher in the OBI/HCV group when compared to the controls group, and no significant difference was found for IFN-Y (*p* = 0.086). However, when comparing the mean values of the measured cytokines, we found that unlike the median, the mean values of IL-10 (*p* = 0.031) were higher in OBI/HCV group, and no significant difference was found for IL-2 (*p* = 0.076) and TNF-α (*p* = 0.133).

**Fig. 1:**
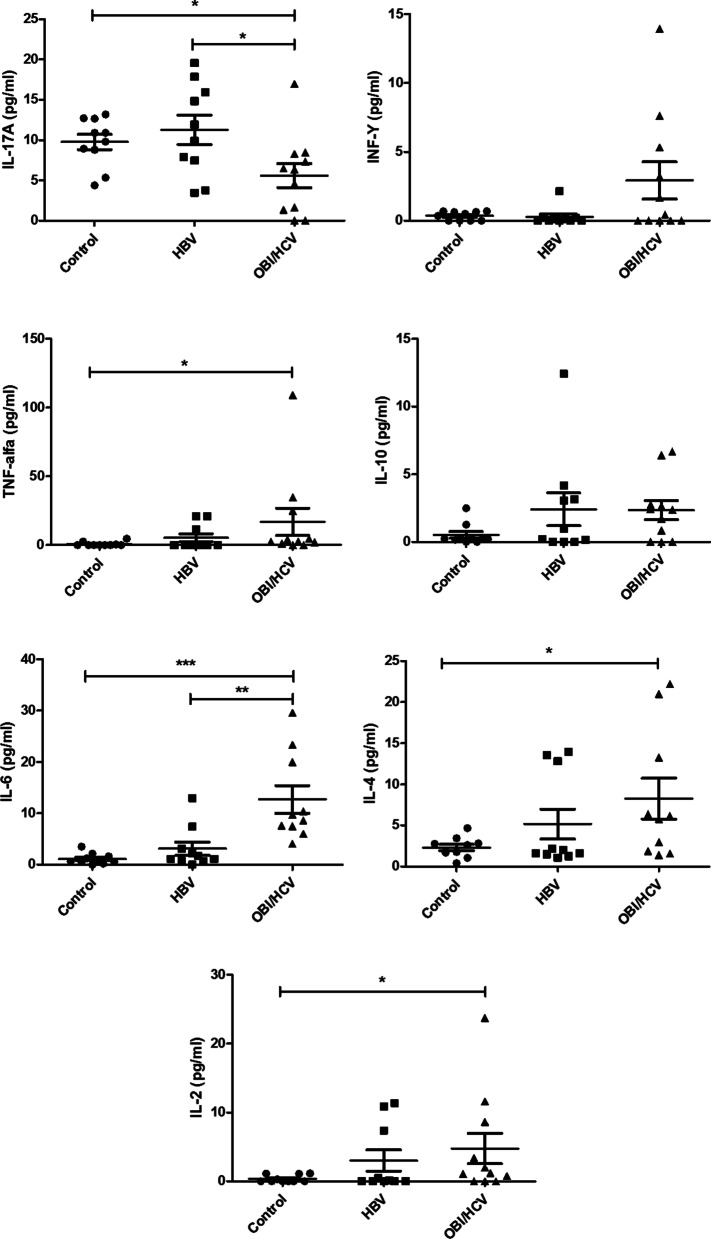
Expression medians levels of inflammatory cytokines in serum from various clinical states of hepatitis B virus infection and controls. Mann Whitney non-parametric test was used to analyze the differences between the groups. *p < 0.05; **p = 0.001 ***p < 0.0001

We found higher medians of IL-17-A in the HBV monoinfected group; a significant difference was found for IL-17A (*p* = 0,0246) between the OBI/HCV and HBV monoinfected groups. A significant difference was found for IL-6 (*p* = 0,0011) between the OBI/HCV and HBV monoinfected groups and no significant difference was found between HBV monoinfected and controls for all analyzed cytokines (Fig. 1 and Table 3).

**Table 3:** Mean ± standard deviation (pg/mL) values of cytokines in each group

	IL-17A	IFN-Y	TNF-α	IL-10	IL-6	IL-4	IL-2
OBI/HCV group	5.58 ± 4.96^a,b^	2.92 ± 4.46	16.87 ± 32.61	2.34 ± 2.33^a^	18.99 ± 22.39^a,b^	11.63 ± 13.43^a^	4.76 ± 7.35
Control group	9.76 ± 3.00^a^	0.35 ± 0.32	0.78 ± 1.60	0.53 ± 0.78^a^	1.12 ± 1.06^a^	2.31 ± 1.22^a^	0.37 ± 0.53
HBV group	11.26 ± 5.69^b^	0.28 ± 0.65	5.32 ± 8.97	2.41 ± 3.84	3.11 ± 4.04^b^	5.16 ± 5.72	3.01 ± 4.83

To know the difference between the groups, the Mann–Whitney nonparametric test was used. Equal letters show groups with statistically significant mean difference

The correlation between cytokines products were analyzed to understand the possible relationship between them. It was possible to observe that there is a positive correlation between cytokines, IFN-Y vs TNF-α (*p* = 0.041), IFN-Y vs IL-10 (*p* = 0.032), IFN-Y vs IL-4 (*p* = 0.013), IFN-Y vs IL-2 (*p* = 0.003), TNF vs IL-2 (*p* = 0.003) and IL4 vs IL-2 9 (*p* = 0.002). The correlations found are classified as moderate to strong. No significant correlations were observed for IL-17A and IL-6 (Table 4).

**Table 4:** Correlation between cytokines in OBI/HCV group through Spearman's correlation coefficient

	IFN-Y	TNF-α	IL-10	IL-4	IL-2
IFN-Y		rô = 0.621 *p* = 0.041	rô = 0.645 *p* = 0.032	rô = 0.715 *p* = 0.013	rô = 0.808 *p* = 0.003
TNF-α			rô = 0.267 *p* = 0.428	rô = 0.888 *p* = 2.57	rô = 0.795 *p* = 0.003
IL-10				rô = 0.294 *p* = 0.381	rô = 0.389 *p* = 0.237
IL-4					rô = 0.826 *p* = 0.002

## Discussion

Antiviral immune responses in OBI are continuously stimulated by persistent/intermittent low concentrations of HBV antigens and cytokines can play an important role in controlling HBV replication [6]. In this present study, the levels of cytokines in OBI/HCV patients were heterogeneous, showing dissemination over a wide range of values with high standard deviations, which can be attributed to the different genotypes and a wide range of viral loads, however, due to the low number of samples, it was not possible to confirm this correlation between genotypes, viral load and the cytokines studied. In the present study, the prevalence of OBI in patients with CHC was 9.6%. Previous studies analyzing serum samples of Brazilian HCV infected patients found frequencies of OBI ranging from 0 to 24% [40–46].

OBI patients usually have a low viral load with suppression of HBV replication and thus, most OBI patients have normal liver histology or minimal fibrosis. However, they are still at risk of developing liver cirrhosis. The prevalence of OBI in cirrhotic patients varies widely from 4 to 38% between different regions of the world [22, 47].

The persistence of HBV DNA in hepatocytes triggers a constant immune response, inducing mild but continuous liver inflammation, which can accelerate liver damage and favor the development of liver cirrhosis in other chronic liver diseases, such as in patients with chronic hepatitis C [22, 48–50]. OBI can contribute to the development of HCC under direct and indirect mechanisms similar to those of chronic HBV infection [51].

In addition, the presence of OBI/HCV coinfection is believed to have an adverse effect on the response to treatment in IFN-based therapies [52]. Coinfection with OBI/HCV was associated with decreased intrahepatic expression of interferon receptor mRNA (IFNAR2), higher levels of serum HCV RNA and a poor IFN response, regardless of the HCV genotype. These results suggest the possibility that coinfection is one of the factors that can lead to an unfavorable IFN response in chronic hepatitis C by negative regulation of the IFN receptor gene expression in the liver [53].

Levels of alanine aminotransferase (ALT) are generally slightly higher in occult hepatitis B in HCV co-infection, with averages ranging from 39 to 158 IU/mL [54, 55]. In our study we found a mean of 62.91 ALT IU/ml, but no correlation was found between the levels of ALT/AST at the age, viral load and the analyzed cytokines.

ALT levels correlate well with inflammatory activity on biopsy, which is generally mild [56]. However, in contrast to this mild inflammation, most studies have shown an increased prevalence of fibrosis/advanced cirrhosis in patients with occult hepatitis B [56–58]. These findings corroborate those of the present study, in which 81.8% of OBI/HCV patients demonstrated stage F4 of fibrosis.

There are few studies on cytokine production profile in occult hepatitis B patients and the mechanism of liver injury due to OBI is still unclear, but some studies describe that the persistence and transcription of HBV cccDNA in hepatocytes can lead to the production of cytokines, such as TNF-α and INF-γ, which can result in damage for hepatocytes [59, 60]. A reduction in proinflammatory cytokines, such as TNF-α, was observed in patients who resolved HBV infection when compared to healthy individuals [59], and IFN-γ was noticeably decreased, especially in monoinfected patients with HCV genotype 1b [61]. In our study, we found higher medians for TNF-α in the OBI/HCV group and no significant difference was found between groups for INF-Y. Most of the patients in our study show stage F4 of fibrosis, which indicates very advanced fibrosis, occult HBV together with the HCV could be contributing to the increase of these cytokines and aggravating the fibrosis condition in these individuals. It is well known that TNF-α is involved with liver inflammation and hepatocytes injury and mediates viral hepatitis complications [32]. However, in this study, the stage F4 cannot be attributed only to OBI, the presence of HCV/HBV infection can be an important role in the advanced fibrosis.

In our study, we found a significant increase in the median of IL-2 in OBI/HCV patients when compared to healthy controls, other studies find overexpression of IL-2 exclusively in patients infected with OBI when compared to healthy individuals and patients who have resolved HBV infection [62] and in patients monoinfected with HCV [63]. IL-2 plays an important role in the efficient development of effector cytotoxic CD8 + T cells, effector cells with a high expression of receptors for IL-2 (IL-2R) are cells that cause direct damage to the liver [64].

Unfortunately, in our study, was not possible to evaluate the cytokines levels in patients monoinfected with HCV. However, a study conducted by Baskic and collegues, 2017 [65] investigating the cytokine profile in chronic hepatitis C demonstrated that median levels of IL-17A were lower in patients with HCV than in controls. In our study, low levels of IL-17A were also found in OBI/HCV coinfected patients. We found significant difference between OBI/HCV compared to monoinfected HBV group and healthy controls with higher means in the monoinfected patients with HBV. The roles of IL-17A in inducing appropriate immune responses against viral infections are controversial, IL-17 may play a positive role in antiviral immune responses in several diseases [66]. However, for chronic hepatitis B, is well known, that IL-17A is positively regulated in HBV-mediated inflammation and may be relevant for the development of liver cirrhosis and HCC [36, 67, 68]. IL-17A can also significantly stimulate monocytes and DCs to express their ligand (IL-17R) and produce pro-inflammatory cytokines such as IL-1β, TNF-α, IL-6, etc., which are important for liver damage during progression of chronic hepatitis B [69].

Other cytokines possibly involved in OBI include increased interleukin 10 (IL-10), IL-10 can leads to reduced expression of IL-12, stromal cell-derived factor (SDF)-1α, and C–C chemokine receptor (CCR), which leads to the interruption of T and natural killer cells (NK cell) activation and the recruitment of immune cells to the infected liver [70]. IL-10 production is also increased in monoinfected patients with chronic hepatitis C, the HCV RNA load is closely associated with IL-10 expressions, and inhibition of HCV replication was accompanied by a reduction in IL-10 [41, 61]. Corroborating these findings, our study showed significantly higher means in IL-10 levels in patients with OBI/HCV compared with controls.

In vivo levels of IL-6 have been associated with plasma ALT levels and the degree of liver fibrosis in patients with HCC in HCV monoinfected patients [71] and a previous study demonstrated a low detection rate of IL-6 in patients infected with OBI when compared to healthy individuals and patients who resolved HBV infection [62]. We found high levels of IL-6 in the OBI/HCV group in our study and significant difference between OBI/HCV compared to monoinfected HBV group and healthy controls, this increase in IL-6 expression could be attributed to HCV co-infection, since several studies have already found that this cytokine is associated with HCV chronic infection [63, 72, 73].

It is well known that IL-6 can play two important roles in the pathogenesis of hepatitis B, can protect the liver from virus infections by stimulating immune responses against infected hepatocytes and can inhibit the HBV entry in hepatocytes up to 90% when cells are treated with IL-6 resulting in a marked reduction in cccDNA and HBsAg secretion [74]. But the IL-6 can also play an important role in the induction of hepatitis, cirrhosis, and HCC [33, 75].

We found levels of IL-4 significantly increased in OBI/HCV patients. IL-4 is a cytokine that can suppress the Th1-type response, maintaining persistent HBV replication and promoting immune tolerance [32, 76, 77]. Some studies show that patients with severe hepatitis C had higher levels of IL-4 compared to milder cases [78] and serum IL-4 levels were significantly increased in patients with chronic HCV [79].

Serum HBV DNA levels are typically low in occult hepatitis B, but despite the low rate of replication, detection of HBV DNA can be associated with ALT outbreaks that can be associated with advanced fibrosis/cirrhosis. In addition, occult OBI/HCV coinfection is associated with decreased response to interferon [53].

There were some limitations in the present study. The biggest limitation was the low number of patients with OBI, but the most of studies with OBI and cytokines analyses from 12 to 30 samples [59, 62, 80]; the second limitation was that there are few studies on the cytokine production profile in patients with OBI/HCV and finally, unfortunately, we could not include monoinfected HCV patients in our analyzes. However, our study was strongly compared to published data and can contribute to a better understanding of the complex response process related to cytokine production in OBI patients coinfected with HCV.

## Conclusion

In conclusion, the results of the present study suggest that there is a significant difference in the detection of IL-17A, TNF-α, IL-10, IL-6, IL-4 and IL-2 in OBI/HCV patients when compared to healthy controls or HBV monoinfected. We found a high level of IL-17A in the HBV monoinfected group and high levels of TNF-α, IL-10, IL-6, IL-4 and IL-2 in OBI/HCV patients. However, further studies are needed to better understand the complex regulatory mechanisms of the host inflammatory response related to cytokine production during OBI/HCV coinfection and to understand the differences in mechanisms underlying infection resolution or the establishment of virus persistence. The expression of distorted cytokines exists in OBI patients coinfected with HCV and the exploration of this pattern of cytokine expression can help to develop a better understanding of the pathogenesis of chronic coinfection by OBI and HCV.

## Supplementary information

**Additional file 1**. Data file of clinical and epidemiological information of patients with occult hepatitis B and HCV enrolled in this study. SAH (Subarachnoid hemorrhage); HCC (Hepatocellular carcinoma); IFN-Peg (Pegylated interferon); HBV (Hepatitis B virus); HCV (Hepatitis C virus); HAV (Hepatitis A virus); HIV (human immunodeficiency virus); IgG (Immunoglobulin G); HBsAg (Hepatitis B virus surface antigen); HBc (Antigen “c” or core of the hepatitis B virus); AST (alanine aminotransferase); ALT (alkaline phosphatase); GGT (gamma-glutamyl transferase); TGO (oxalacetic glutamic transaminase); TGP (glutamic-pyruvic transaminase); ALB (albumin); AFP (alpha-fetoprotein); LDL (Low Density Lipoproteins); HDL (High Density Lipoproteins); HGB (Hemoglobin); A.T (After treatment)

## Data Availability

The dataset is available from the corresponding author.

## Abbreviations

HBV: Hepatitis B virus
OBI: Occult hepatitis B
IFN-γ: Interferon gamma
TNF: Tumor necrosis factor
IL: Interleukin
HCV: Hepatitis C virus
DNA: Deoxyribonucleic acid
HCC: Hepatocellular carcinoma
cccDNA: Covalently closed circular DNA
HBsAg: Hepatitis B surface antigen
Anti-HBc: Antibody against the viral core
DAA: Direct-acting antivirals
EIA: Immunoenzymatic assays
PCR: Polymerase chain reaction
qPCR: Real-time PCR
AST: Aminotransferase
Alanine ALT: Aminotransferase
GGT: Gamma-glutamyl transferase
CIs: Confidence intervals
SD: Standard deviation
ND: Not determined
(SDF)-1α: Stromal cell-derived factor
CCR: C–C chemokine receptor
NK cell: Natural killer cells
